# Adult Descending Colon Duplication Mimicking Colon Cancer: A Rare Case Report

**DOI:** 10.70352/scrj.cr.26-0089

**Published:** 2026-04-24

**Authors:** Shosaburo Oyama, Shuichi Tobinaga, Kazuto Shigematsu, Yoshihito Shibata

**Affiliations:** 1 Department of Gastroenterological Surgery, Japanese Red Cross Nagasaki Genbaku Hospital, Nagasaki, Nagasaki, Japan; 2 Department of Pathology, Japanese Red Cross Nagasaki Genbaku Hospital, Nagasaki, Nagasaki, Japan

**Keywords:** adult, intestinal duplication, descending colon, colon cancer, laparoscopic colectomy

## Abstract

**INTRODUCTION:**

Intestinal duplication is a rare congenital anomaly characterized by the presence of a segment of intestinal tissue containing both mucosal and muscular layers adjacent to the normal alimentary tract. Intestinal duplication is most commonly diagnosed in infancy and is rarely observed in adults. Colonic duplications are particularly uncommon and may be difficult to distinguish from malignancy. Herein, we report a rare case of an adult descending colon duplication that was suspected preoperatively to be colon cancer.

**CASE PRESENTATION:**

A 75-year-old man with a history of surgery for prostate cancer and a left inguinal hernia was referred to our department of gastroenterological surgery. Preoperative abdominal CT revealed a circumferential mass in the descending colon. Colonoscopy revealed a circumferentially elevated lesion approximately 30 cm from the anal verge with luminal narrowing that prevented scope passage. A biopsy revealed no malignancy. Owing to intestinal stenosis caused by the mass and suspicion of malignancy, we performed a laparoscopic left hemicolectomy with D3 lymph node dissection. Intraoperatively, the mass was adhered to 4 segments of the small intestine, necessitating partial small bowel resection. Histopathological examination revealed a tubular structure on the serosal side of the native colon lined with colonic-type mucosa and possessing its own muscularis propria, continuous with the normal colonic wall. These findings were consistent with intestinal duplication. The postoperative course was uneventful, and the patient was discharged on the 13th POD. No recurrence was detected on follow-up CT at 18 months postoperatively.

**CONCLUSIONS:**

Colonic duplication in adults is extremely rare and may mimic colorectal carcinoma on imaging and endoscopy. Surgical resection provides both definitive diagnosis and curative treatment.

## Abbreviations

CA 19-9
: carbohydrate antigen 19-9
CEA
: carcinoembryonic antigen
CRP
: C-reactive protein
EUS
: endoscopic ultrasonography
WBC
: white blood cell

## INTRODUCTION

Colonic duplication is a rare congenital anomaly, accounting for 5%–15% of intestinal duplications, and most commonly occurs in the ileum or jejunum. Cases involving the descending colon are particularly rare. Furthermore, most intestinal duplications are detected during early childhood, with the majority diagnosed before 2 years of age.^1–4)^

Adult-onset intestinal duplication syndrome often presents without specific symptoms beyond abdominal pain, gastrointestinal bleeding, perforation, or intestinal obstruction, and is frequently discovered incidentally. Preoperative diagnosis of intestinal duplication syndrome is also considered difficult. As in the present case, tumor-like lesions are often incidentally detected on imaging studies or endoscopy.^4–6)^ In most cases, lesion resection is performed for diagnostic or therapeutic purposes, which leads to a definitive postoperative diagnosis. Additionally, there are reports in which malignant tumors such as colorectal cancer have been identified in resected specimens, supporting the justification for surgical intervention.^5–8)^

Herein, we report a case in which laparoscopic radical resection was successfully performed for descending colon duplication with suspected colorectal cancer in an adult patient.

## CASE PRESENTATION

**Fig. 1:**
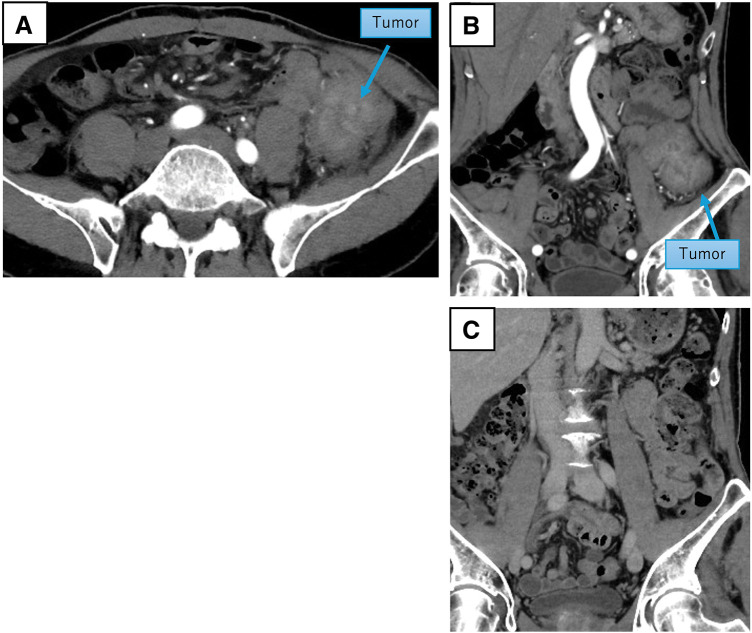
Preoperative abdominal contrast CT. Preoperative images show a 50 × 50 × 60-mm mass (blue arrows) with wall thickening in the mid-descending colon, raising the suspicion of infiltration into the adjacent small intestine and abdominal wall (**A**, **B**). A CT image (**C**) obtained 1 year prior to prostate surgery, demonstrating no masses in the descending colon.

**Fig. 2:**
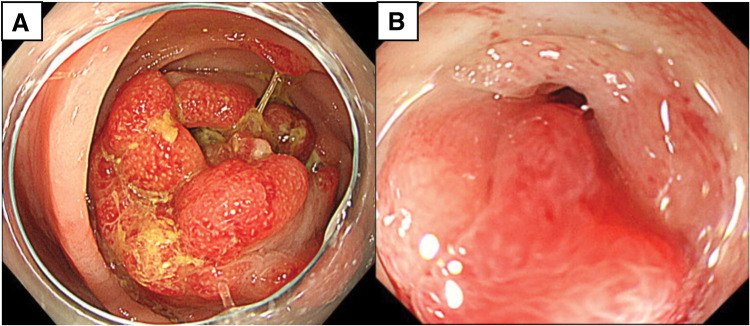
Preoperative colonoscopy. Preoperative colonoscopy shows a mass located 30 cm from the anal verge in the middle of the descending colon (**A**), causing significant luminal narrowing, which prevented passage of the scope (**B**).

A 75-year-old man with a history of surgery for prostate cancer 1 year prior to presentation was admitted to our hospital’s urology department. He was referred to our department of gastroenterological surgery for the treatment of a left inguinal hernia. The patient reported few symptoms; although he had been experiencing a sense of discomfort in his lower left abdomen for several months, no significant symptoms were noted. Apart from the diagnosis of a left inguinal hernia, no obvious abnormalities such as a mass were found on abdominal examination. Preoperative abdominal CT incidentally revealed a circumferential mass-like lesion in the descending colon (**Fig. 1**). The mass was 52 × 50 × 60 mm with wall thickening in the mid-descending colon, raising suspicion of infiltration into the adjacent small intestine and abdominal wall. A CT image (**Fig. 1C**) acquired 1 year prior to prostate surgery demonstrated no tumorous lesions in the descending colon. Although the CT image did not reveal intestinal obstruction, the colon proximal to the mass showed signs of fecal impaction. Colonoscopy showed a circumferentially elevated lesion located approximately 30 cm from the anal verge, causing significant luminal narrowing that prevented passage of the scope (**Fig. 2**). The mucosal surface of the mass was erythematous and caused narrowing of the lumen; however, no findings suggestive of a malignant tumor were observed on biopsy. Furthermore, based on the CT scan, a massive neoplastic lesion was suspected, and given the severe stenosis, subileus was suspected; therefore, additional examinations such as a barium enema, EUS, and MRI were not performed. Preoperative blood test results showed a WBC count of 6700/μL (with a neutrophil percentage of 36.8%) and CRP level of 0.28 mg/dL; no signs of inflammation were observed. The tumor markers—CEA at 1.5 ng/mL and CA19-9 at 30.6 U/mL—were within the normal range. Although a definite diagnosis was not made, malignancy could not be excluded. Following a colonoscopy, a decision was made to proceed with early surgery. After 1 week of fasting and intravenous fluid management during hospitalization, the scheduled early surgery was performed.

**Fig. 3:**
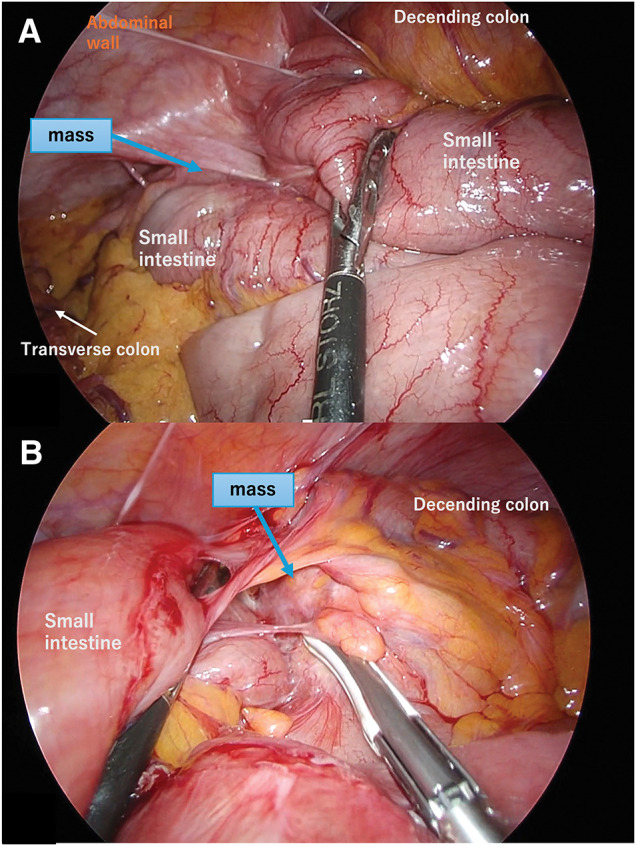
Intraoperative laparoscopic view. Intraoperative findings confirm that the mass was located in the mid-descending colon, as demonstrated by preoperative imaging, with adhesions suggesting possible infiltration of the adjacent abdominal wall (**A**) and small bowel (**B**). Based on these findings, we performed a laparoscopic left hemicolectomy (D3) along with partial small bowel resection.

**Fig. 4:**
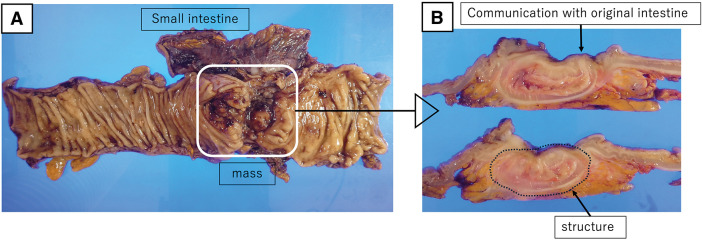
Extracted specimen. In the excised specimen, a tubular structure continuous with and communicating with the original intestine was observed.

We performed a laparoscopic left hemicolectomy with D3 lymph node dissection. The procedure began as a 5-port laparoscopic surgery; once complete mobilization of the left colon and vascular management were completed, the umbilical port site was converted into a 5-cm small open incision. The mass and its adhesions to the small intestine were extracted as a single mass through the small open incision and manipulated extracorporeally. Intraoperative findings revealed dense adhesions between the mass and 4 segments of the small intestine, which were partially resected due to involvement (**Fig. 3**). During the procedure, a tumor-like induration was palpated on the colonic side of the mass, and we could not rule out malignancy. We determined that resection of the infiltrated area was necessary and performed a partial small bowel resection. The operative time was 299 min, and blood loss was 30 mL. Gross examination of the resected specimen revealed a mass running parallel to the descending colon. The lesion communicated with the colonic lumen (**Fig. 4**).

**Fig. 5:**
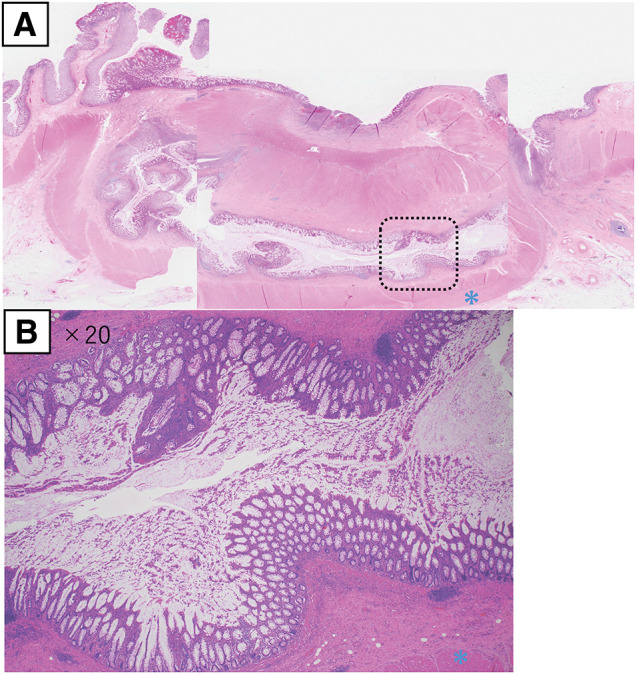
Histopathological findings. Histopathological examination of hematoxylin and eosin-stained tissue demonstrates a tubular structure containing intestinal mucosa and muscularis propria (blue asterisks). Communication with the original intestine is also shown. There was no evidence of dysplasia or malignancy. (**A**) Low-power view and (**B**) ×20 view of the region indicated by the dashed-line box in (**A**).

Histopathological examination demonstrated a tubular structure containing intestinal mucosa and muscularis propria. Communication with the original intestine was also identified. There was no evidence of dysplasia or malignancy (**Fig. 5**). Based on these findings, the patient was diagnosed with a tubular-type descending colon duplication. The patient’s postoperative course was uneventful, and he was discharged 13 days after surgery. No recurrence was detected on follow-up CT at 18 months postoperatively.

We speculate that localized infection within the duplicated segment caused inflammatory thickening, resulting in mass formation and compression of the native colon, thereby mimicking a malignant stricture.

## DISCUSSION

Alimentary duplication, classically defined by Ladd and Gross,^9)^ is a rare congenital anomaly characterized by a structure that is intimately associated with the alimentary tract, lined by gastrointestinal mucosa, and possesses a well-developed smooth muscle layer. Morphologically, duplications are classified into cystic and tubular types and communication with the adjacent intestinal lumen may or may not occur. Depending on the location of duplication, infiltration into the urinary tract, genitalia, or surrounding skeletal muscles is possible.^3,4)^ According to various reports, cystic and tubular intestinal duplication types account for 80% and 20%, respectively,^1,2,10)^ whereas tubular types are more common in colonic or rectal duplications.^3,6,9)^ Furthermore, most cases of intestinal duplication present with symptoms during early childhood, typically before the age of 2. The most common site of intestinal duplication is the ileum, followed by the jejunum and colon.^1–4)^ Therefore, descending colon duplication in adults, as observed in the present case, is extremely rare.

In this study, we searched PubMed and Igaku Chuo Zasshi using the keywords ‘‘colon duplication’’ and ‘‘adult’’ to identify cases from 1996 to 2025 that were available and suitable for analysis. We defined adult cases as those aged 15 years or older and excluded cases involving the rectum or entire colon, which often present with concomitant congenital anomalies. Including our own case, the total number of cases was 74 (**Table 1**). The median age was 38.5 years, and the sex ratio showed a slight tendency toward females, accounting for 52.7%. There were no specific symptoms, and the descending colon accounted for 8.1% (6/74 cases), which was low among the colonic duplications. While previous reports indicate that the preoperative diagnosis for intestinal duplication was low at around 11.2%,^11,12)^ in this study, the rate for colonic duplication was 34.2% (25/74 cases), which was higher than previously reported. We consider this may be because lesions in the colon can be evaluated more directly compared to the small intestine, through procedures such as colonoscopy or barium enema, and because communication between the lesion and adjacent intestinal segments is relatively common, making barium enema an effective diagnostic tool. In this case, the condition was judged to be on the borderline of intestinal obstruction during colonoscopy, and barium enema could not be performed due to concerns that it might worsen the patient’s general condition.

**Table 1:** Characteristics of 74 published cases of colon duplication including our case

Characteristics of colon duplication in adults	
Median age at onset (years) (range)	38.5 (16–81)
Sex, male (%)/female (%)/NA	34 (45.9%)/39 (52.7%)/1
Clinical symptom	
Asymptomatic	7
Abdominal pain, acute/intermittent and discomfort	12/38
Constipation/intestinal obstruction/intussusception	12/3/6
Hematochezia/perforation/intraabdominal abscess	3/3/3
Tumor size (mm), median maximum diameter (range)	360 (15–720)
Location	
Appendix/cecum/ascending colon	3/11/18
Transverse colon/descending colon/sigmoid colon	21/6/15
Preoperative diagnosis, yes (%)/no (%)	25 (34.2%)/48 (65.8%)
Preoperative diagnosis of a suspected solid tumor	13 cases
Surgery	
Emergency surgery/elective surgery/no surgery/NA	15/55/3/1
Laparotomy/laparoscopic/no surgery (endoscopic)/NA	43/25/1/1
Morphological types, cystic/tubular	41/33
Communication with the adjacent intestinal tract, yes/no/NA	49/18/7
Presence of malignant tumor, yes (%)/no (%)/NA	6 (8.1%)/67 (90.5%)/1

NA, not available

Adult colonic duplications may present with nonspecific symptoms such as abdominal distension,^13)^ abdominal pain,^4,6,8)^ abdominal masses,^4–6,13)^ or bowel obstruction.^7)^ Although extremely rare, some cases are identified due to chronic constipation.^1,5,14,15)^ Consequently, preoperative diagnosis is challenging, and the condition is sometimes detected incidentally on CT or MRI as a cystic or solid mass adjacent to the colon or intestinal tumor.^1,4,6,8,16,17)^ In the present case, the lesion was discovered incidentally during preoperative imaging for an unrelated condition. When the lesion causes luminal narrowing or mucosal thickening, it may mimic colorectal cancer. Colonoscopy may show extramural compression or circumferential lesions; however, biopsies often yield normal mucosa because the duplication is submucosal or extramural. A definitive diagnosis is usually made based on histopathology after surgical resection.

Surgical excision is generally recommended to prevent complications such as infection, bleeding, perforation, or rarely, malignant transformation.^7,8)^ Colonic duplications have been reported to carry a higher risk of cancerous changes than other intestinal duplications.^7)^ In the present case, surgical resection was necessary for diagnosis and potential malignancy treatment. Various surgical approaches for intestinal duplications have been reported, including recent reports on robotic surgery.^18–20)^ Laparoscopic surgery is useful for intestinal duplications because it allows for access from multiple angles and is suitable for cases like this one involving small-bowel adhesions. In the present case, the laparoscopic approach was considered safe and effective.

The present case likely involved an infection within the duplicated segment, which triggered abscess formation and surrounding inflammation, ultimately leading to mass formation. Based on the clinical course, it was not possible to identify a specific episode that clearly marked the onset of infection. The sensation of discomfort in the lower left abdomen might be consistent with chronic, persistent inflammation. Preoperative blood test results showed no signs of inflammation; therefore, even if inflammation was present in this case, it is presumed to have been chronic.

As noted by Nakahara et al.^21)^ and Akita et al.^22)^ it is presumed that chronic inflammation within a blind-ended duplicated intestinal segment was caused by chronic irritation resulting from secretions from adjacent intestinal segments and fecal stasis. There were 13 cases in which the segment had become tumor-like, as in the present case. It is speculated that relatively small, infected duplicated intestinal segments may lead to thickening of the surrounding intestinal wall or formation of a tumor-like mass due to the spread of inflammation. Also, there are reports suggesting that intestinal duplication may carry a high risk of malignant transformation due to chronic inflammation.

In this study, excluding the 2 cases^23,24)^ in which malignant tumors were identified preoperatively, there were 13 cases^6,8,25–34)^ with solid tumor-like masses prior to surgery, and malignant tumors were confirmed in 4 of those cases.^8,26–28)^ The overall incidence of malignant tumors in this study was 8.1% (6/74 cases), which is not a small number. Therefore, we consider that when colonic duplication presents with mass formation, treatment should be approached with strong awareness of the possibility of a malignancy.

## CONCLUSIONS

Adult colonic duplication is an extremely rare congenital anomaly. Because imaging and endoscopic findings may mimic colorectal carcinoma, it should be considered in the differential diagnosis of atypical colonic masses. Surgical resection provides both a definitive diagnosis and curative management.

## DECLARATIONS

### Availability of data and materials

The datasets used or analyzed during this study are available from the corresponding author upon reasonable request.
